# Morbidity and mortality risks associated with valproate withdrawal in young adults with epilepsy

**DOI:** 10.1093/brain/awae128

**Published:** 2024-04-24

**Authors:** Gashirai K Mbizvo, Tommaso Bucci, Gregory Y H Lip, Anthony G Marson

**Affiliations:** Liverpool Centre for Cardiovascular Science at University of Liverpool, Liverpool John Moores University and Liverpool Heart and Chest Hospital, Liverpool, L69 7TX, UK; Pharmacology and Therapeutics, Institute of Systems, Molecular and Integrative Biology, University of Liverpool, Liverpool, L69 7BE, UK; Neurology Service, The Walton Centre NHS Foundation Trust, Liverpool, L9 7LJ, UK; Liverpool Centre for Cardiovascular Science at University of Liverpool, Liverpool John Moores University and Liverpool Heart and Chest Hospital, Liverpool, L69 7TX, UK; Department of General and Specialized Surgery, Sapienza University of Rome, Rome, 00185, Italy; Liverpool Centre for Cardiovascular Science at University of Liverpool, Liverpool John Moores University and Liverpool Heart and Chest Hospital, Liverpool, L69 7TX, UK; Department of Clinical Medicine, Danish Centre for Health Services Research, Aalborg University, Aalborg, 9220, Denmark; Pharmacology and Therapeutics, Institute of Systems, Molecular and Integrative Biology, University of Liverpool, Liverpool, L69 7BE, UK; Neurology Service, The Walton Centre NHS Foundation Trust, Liverpool, L9 7LJ, UK

**Keywords:** men’s health, women’s health, reproductive age, pharmacology, anticonvulsants, valproic acid

## Abstract

Valproate is the most effective treatment for idiopathic generalized epilepsy. Current guidance precludes its use in females of childbearing potential, unless other treatments are ineffective or not tolerated, because of high teratogenicity. This risk was recently extended to males. New guidance will limit use both in males and females aged <55 years, resulting in withdrawal of valproate from males already taking it, as occurs for females. Whether there are risks of personal harm (including injury or death) associated with valproate withdrawal has not yet been quantified for males or females ON valproate, meaning clinicians cannot reliably counsel either sex when discussing valproate withdrawal with them, despite that this concern may be at the forefront of patients’ and clinicians’ minds. We assessed whether there are any morbidity or mortality risks associated with valproate withdrawal in young males and females.

We performed a retrospective cohort study of internationally derived electronic health data within the TriNetX Global Collaborative Network. Included were males and females aged 16–54 years with ≥1 epilepsy disease or symptom code between 1 December 2017 and 1 December 2018, and ≥2 valproate prescriptions over the preceding 2 years (1 January 2015–30 November 2017). Five-year propensity-matched risks of mortality and a range of morbidity outcomes were compared between those remaining ON versus withdrawn from valproate during the 1 December 2017–1 December 2018 recruitment period, regardless of whether switched to another antiseizure medication. Survival analysis was undertaken using Cox-proportional hazard models, generating hazard ratios (HRs) with 95% confidence intervals (CIs).

In total, 8991 males and 5243 females taking valproate were recruited. Twenty-eight per cent of males and 36% of females were subsequently withdrawn from valproate. Valproate withdrawal was associated with significantly increased risks of emergency department attendance [HRs overall: 1.236 (CI 1.159–1.319), males: 1.181 (CI 1.083–1.288), females: 1.242 (CI 1.125–1.371)], hospital admission [HRs overall: 1.160 (CI 1.081–1.246), males: 1.132 (CI 1.027–1.249), females: 1.147 (CI 1.033–1.274)], falls [HRs overall: 1.179 (CI 1.041–1.336), males: 1.298 (CI 1.090–1.546)], injuries [HRs overall: 1.095 (CI 1.021–1.174), males: 1.129 (CI 1.029–1.239)], burns [HRs overall: 1.592 (CI 1.084–2.337)] and new-onset depression [HRs overall 1.323 (CI 1.119–1.565), females: 1.359 (CI 1.074–1.720)]. The risk of these outcomes occurring was 1%–7% higher in those withdrawn from valproate than in those remaining ON valproate. Overall, valproate withdrawal was not associated with increased mortality.

These results may help patients and clinicians have a more informed discussion about personal safety when considering valproate withdrawal.

## Teaser

Valproate effectively controls seizures in epilepsy, but there are restrictions on its use in males and females aged <55 years because of its high teratogenicity. Mbizvo *et al.* show using electronic health data that there may also be risks of personal harm to the males and females who have valproate discontinued.

## Introduction

Epilepsy is one of the most common neurological disorders, affecting more than 70 million people worldwide and accounting for 0.5% of the global burden of disease.^1^ Valproate is one of the most effective drugs for treating epilepsy, outperforming any other in idiopathic generalized epilepsy [which affects a third of people with epilepsy (PWE)].^2-4^ Guidance introduced between 2015 and 2018 prohibits its use in females of childbearing potential with epilepsy unless other antiseizure medications (ASMs) are ineffective or not tolerated.^5,6^ This is because valproate exposure during pregnancy is associated with a substantial risk of foetal congenital malformations (11%)^7^ and neurodevelopmental disorders (30%–40%),^5,6^ frequently leading to permanent disability (∼17 000–30 000 births affected since Sanofi first marketed the drug in 1967).^8-10^ This risk was recently extended to males following the discovery of cumulative risks of 5.6%–6.3% for neurodevelopmental disorders and also of transgenerational gene expression alterations in the offspring of males taking valproate.^8,11^ Reduced male fertility is also reported, reversible upon valproate discontinuation.^12,13^ Plans for changes to valproate prescribing were announced in December 2022 by the Commission on Human Medicines.^8^ These will prohibit valproate prescribing for both males and females aged <55 years unless other treatments are ineffective or not tolerated.^8^ The measures will be rolled out nationally over the coming months in the UK,^8,14^ with similar measures being considered in Europe,^15^ New Zealand^11^ and Singapore.^16^ This will result in withdrawal of valproate from many males already taking it, as has been the case for females taking it since 2015–18.^8^ Whether there are any potential risks of personal harm or morbidity (including hospital admissions, injuries, depression or death) associated with valproate withdrawal has not yet been quantified for males or females ON valproate.^17-30^ This means patients currently cannot have a fully informed discussion about their personal safety with clinicians when discussing valproate withdrawal with them,^31,32^ despite that this concern may be at the forefront of patients’ and clinicians’ minds.^33-36^ So far, studies assessing valproate withdrawal have tended to focus on the group of PWE in remission from seizures for at least 2 years, usually showing worsening seizure control in generalized epilepsy. They have tended to be single-centre studies or small, looking at seizures alone as the outcome of interest, or assessing females alone.^17,18,21-29^

We performed an international cohort study aimed at describing whether there are any wider morbidity or mortality risks associated with valproate withdrawal (regardless of whether or not switched to another ASM) for males and females aged 16–54 years with epilepsy. The results will help the males and females with epilepsy who may need to have valproate withdrawn for fertility or teratogenicity reasons have a more fully informed discussion about this with their clinician.

## Materials and methods

### Study design and setting

We undertook a retrospective cohort study of international data in the TriNetX research platform. TriNetX is the world’s largest ecosystem of real-word electronic health data, drawn from ∼250 million patients from >120 healthcare organizations (HCOs) across 19 countries predominantly in North America but also South America, Europe, the Middle East, Africa and Asia Pacific.^37-39^ It holds ∼70 billion date- and patient-indexed electronic clinical observations.^37^ Much of the data imported by TriNetX come from large academic HCOs, although data from smaller rural hospitals are also imported to better reflect patients from all care backgrounds. Similarly, recruitment of HCOs into the TriNetX platform is agnostic of insurance status, meaning data from both insured and uninsured patients are captured. Data are imported from a variety of healthcare settings including emergency care, inpatients, outpatients and primary care. The data imports occur in real-time from HCOs that are online at the time a researcher connects to the TriNetX network and undertakes a search, meaning results are contemporaneous with frontline clinical progress. Variables held include demographics, diagnoses [using International Classification of Diseases, 10th Revision, Clinical Modification (ICD-10-CM) codes], drugs (RxNorm-coded), procedures [including current procedural terminology (CPT) and SNOMED codes], laboratory results, genomics, tumour registration and mortality data. The mortality data are enriched with data sourced from outside healthcare settings to increase coverage.^39^ More information about TriNetX can be found online (https://trinetx.com).^37,39^

### Search strategy and participants recruited

We searched the Global Collaborative Network in TriNetX’s study query builder for males and females aged 16–54 years with at least one G40 (epilepsy) or R56.9 (seizure) ICD-10-CM code recorded between 1 December 2017–1 December 2018 and at least two valproate prescriptions over the preceding 2 years (1 January 2015–30 November 2017). Two comparison cohorts were generated: those withdrawn from versus those remaining ON valproate through the 1 December 2017–1 December 2018 recruitment period. Outcome assessment commenced for 5 years thereafter, starting a day after the first epilepsy disease/symptom code to appear without and with co-prescribed valproate during recruitment. No restrictions were placed on other ASMs used as these were propensity-matched at baseline between comparison groups (refer to the ‘Statistical analysis’ section). Three network searches were undertaken on 28 January 2024. The first search applied no filter by sex, allowing import of data from males and females combined. The second search filtered out females, and the third search filtered out males.

### Case-ascertainment accuracy

Studies validating the diagnostic accuracy of ICD-10 coding for epilepsy in TriNetX specifically are as yet unavailable. However, more widely, there are a substantial number of studies validating the diagnostic accuracy of these codes, including in regions and settings covered by TriNetX.^40^ As we showed previously, a case-ascertainment strategy combining disease coding (G40, including epilepsy and status epilepticus) and/or symptom coding (R56.9) with co-prescribed ASMs is a valid way to accurately capture epilepsy within electronic healthcare datasets, and this spans multiple studies in a variety of healthcare settings and countries.^40-43^ This strategy tends to achieve figures of 80%–90% across positive predictive values (PPVs) and sensitivity estimates, and negative predictive values (NPV) and specificity estimates approach 100%.^40-43^ The corresponding F1 scores are also high at 0.86.^43^

### Outcomes

The TriNetX query builder primarily allows for dichotomous assessment of outcomes to enable a time-to-first-event survival analysis approach, although event frequency summaries can also be shown. We compared cohorts for whether or not the coded outcomes listed below occurred during the 5-year observation period. The outcomes were selected from a combination of prior literature^44-48^ and input from members of the public affected by epilepsy working with us as public advisors:

- all-cause death^49-51^;
- one or more all-cause emergency department attendances (HL7v3-coded encounters)^52,53^;
- one or more all-cause hospital admissions (HL7v3-coded encounters)^52,53^;
- one or more seizure-coded healthcare consultations (R56.9-coded)—i.e. seizures coming to the attention of healthcare providers and coded by them^40-43^;
- one or more falls (W00–19-coded)^54^;
- one or more injuries (S–T-coded, see Supplementary material, Section S1 for full list)^55^;
- one or more burns (T20–25/T30–32-coded)^55,56^;
- one or more aspiration pneumonia infections (J69.0/J69.8-coded)^57,58^;
- incident depression (F32.0–32.9, F33.0–33.3, F33.8, F33.9, F34.1 or F41.2 coded without any prior instances in the entire record)^59^; and
- one or more self-harm (X71–83-coded) or suicide attempts (T14.91-coded).^60^

### Outcome-ascertainment accuracy

Although studies validating the diagnostic accuracy of outcome coding in TriNetX specifically are as yet unavailable, the coding strategies we used were developed from prior literature assessing similar outcomes, coding guidelines or relevant diagnostic accuracy studies (as referenced in the list earlier).^40-43,49-60^

### Statistical analysis

The following baseline characteristics were compared and matched between those withdrawn from versus remaining ON valproate. The comparisons were made using chi-squared tests for categorical variables and independent-sample *t*-tests for continuous variables:

- age at the beginning of follow-up;
- ethnic background (categorized centrally by TriNetX into black, white, Asian, American Indian or Alaska Native, Native Hawaiian or Other Pacific Islander, other race, or unknown race);
- sex;
- type of epilepsy (using G40 codes);
- one or more seizure-coded healthcare consultations over the year preceding follow-up (R56.9 codes);
- ASMs used other than valproate over the year preceding follow-up (see Supplementary material, Section S2 for the list of ASMs considered);
- one or more psychiatric comorbidities coded over the year preceding follow-up [F30–39: mood (affective) disorders, F20–29: schizophrenia, schizotypal, delusional, and other non-mood psychotic disorders; F99–99: unspecified mental disorder (F99);
- one or more self-harm (X71–83-coded) or suicide attempts (T14.91-coded) over the year preceding follow-up;
- one or more falls (W00–19-coded) over the year preceding follow-up;
- one or more emergency department attendances (CPT-coded) over the year preceding follow-up; and
- one or more hospital admissions (CPT-coded) over the year preceding follow-up.

Standardized mean differences (std diff.) were used to show the distribution of these baseline characteristics among the cohorts and calculated as the difference in the means or proportions of a particular variable divided by the pooled estimate of standardized differences for that variable. Propensity score matching (PSM) 1:1 was used to minimize any significant differences in baseline characteristics between the two cohorts, aiming for a std diff. of <0.1 to indicate adequate matching.^61^ Cohorts were matched for all of the baseline characteristics listed above prior to follow-up.

Survival analysis was undertaken on the fully matched cohorts (std diff. <0.1) using Cox-proportional hazard regression models to calculate hazard ratios (HRs) with 95% confidence intervals (CIs) for the 5-year risk of each outcome. Kaplan-Meier plots were generated for statistically significant results. Log-rank *P*-values were also shown (<0.05 cut-off). Participants were removed from the analysis (censored) after the last fact in their record. As the occurrence of death would preclude the occurrence of any other outcome through informative censoring, this was the main competing risk to consider. We also considered emergency department attendance and hospital admission as potentially competing risks as these are usually recorded as separate (sequential) care spells, wherein hospital inpatients are precluded from simultaneous emergency department attendance. Competing risk was not a relevant consideration across the remaining outcomes as these were not mutually exclusive. We used the Aalen-Johansen estimator of the cumulative incidence function (CIF) to display the incidence of the occurrence of death against each of the other outcomes. Emergency department attendance and hospital admission were included as separate curves to allow additional visualization between these. The likelihood for death to have inferred a substantial competitive risk against an outcome was considered high if death displayed a higher cumulative incidence than the outcome at the end of follow-up or if the CIF curve for death and the outcome intersected. We also added a composite measure of emergency department attendance and/or hospital admission to the survival analysis empirically to allow for the potentially competing risk between these two outcomes.

The primary analysis combined males and females into a single analysis, matched by sex and the rest of the baseline characteristics. Secondary analyses were undertaken separately in a male and in a female dataset. A tertiary analysis was also planned, where possible, to assess outcomes in females who withdrew from valproate then became pregnant during recruitment (captured using Z33 and O00–09A codes for pregnancy). This was done to help assess outcomes in situations where it is likely females were planning pregnancy and elected to withdraw from valproate in anticipation of this.

All analyses were performed on 28 January 2024 in the TriNetX platform, which uses R’s survival package v3.3.

### Patient and public involvement

Through NIHR Applied Research Collaboration North West Coast (ARC NWC), we collaborated with a public advisor with lived experience of taking valproate, who influenced study design by helping ensure outcomes selected were meaningful for PWE, reviewed drafted protocols for appropriateness, and will assist with disseminating published results amongst the community of people affected by epilepsy. This study is also part of a wider project using electronic health data to investigate and predict morbidity and mortality in epilepsy,^62^ which was presented at an ARC NWC public engagement forum attended by various members of the public affected by epilepsy, who were given an opportunity to feed back on the planned work.

### Ethics statement

The University of Liverpool Research Ethics decision tool was used to determine that ethical approval was not required as the study was a secondary analysis of data that were anonymized by an external party (TriNetX) and provided to the research team in the fully anonymized format.^63^ Further, The University of Liverpool Research Ethics Committee has provided formal ethics review exemption for this study as it does not involve human participants, human tissue or personal data (REC Ref. 14455). Data were de-identified per the de-identification standard defined in Section 164.514(a) of the HIPAA Privacy Rule.^37^ TriNetX data are attenuated to ensure participating HCOs remain anonymized. This includes withholding the names of participating countries with less than three HCOs contributing data.

## Results

A study population of 14 412 PWE aged 16–54 years ON valproate was identified, drawn from a pool of 112 HCO that were online at the time of the searches. These HCOs were predominantly located in the USA (yielding 13 667 PWE, 95% of the study population). Of the remainder, 3% (415 PWE) came from Brazil, Italy, Spain, Taiwan and the UK. To protect patient anonymity, TriNetX shielded the names of the remaining 10 countries contributing the final 2% (330 PWE) as each had less than three HCOs participating. date- and patient-indexed electronic clinical observations (*n* = 66 108 884) were gathered from the study population. Ninety-five per cent of these were recorded between 2008 and 2023. Of the 14 412 PWE ON valproate, 4436 (31%) were subsequently withdrawn from treatment, and 9976 (69%) remained ON treatment. A technical limitation in the data linkage prevented PSM for three people in the group remaining ON valproate, resulting in their exclusion. The remaining 14 409 PWE progressed to PSM. Table 1 shows their baseline demographic and clinical characteristics after PSM (see Supplementary Table 1 for these characteristics before matching). Eight thousand nine hundred and ninety-one were males, 5243 were females and gender was unknown in 175 people (66 withdrawn from valproate, 109 remaining ON valproate). Overall, this was a young study population (mean age 27 years) with predominantly generalized epilepsy. Supplementary Table 2 shows their co-prescribed ASMs before and after PSM. Outcomes were compared between the 4396 matched PWE in each cohort. Those of unknown gender were matched to each other and included in this analysis. They were then excluded when males and females were analysed separately from each other.

**Table 1:** Baseline characteristics after propensity score matching: males and females combined

	4396 withdrawn from valproate (Cohort 1) matched with 4396 remaining ON valproate (Cohort 2)					
Cohort	Characteristic		Patients	% of cohort	*P*-value	Std diff.
1 2	Age at index	27.4 ± 11.5 mean ± SD 27.6 ± 11.4 mean ± SD	4396 4396	100% 100%	0.450	0.016
1 2	Male		2489 2477	56.6% 56.3%	0.796	0.006
1 2	Female		1843 1857	41.9% 42.2%	0.762	0.006
1 2	Unknown gender		64 62	1.5% 1.4%	0.858	0.004
1 2	White		2866 2906	65.2% 66.1%	0.369	0.019
1 2	Black or African American ethnicity		701 715	15.9% 16.3%	0.685	0.009
1 2	Unknown ethnicity		499 483	11.4% 11.0%	0.588	0.012
1 2	Other ethnicity		205 186	4.7% 4.2%	0.326	0.021
1 2	Asian ethnicity		84 66	1.9% 1.5%	0.138	0.032
1 2	American Indian or Alaska Native ethnicity		32 32	0.7% 0.7%	1	<0.001
1 2	Native Hawaiian or other Pacific Islander		10 10	0.2% 0.2%	1	<0.001
1 2	R56.9 Unspecified convulsions		1640 1534	37.3% 34.9%	0.019	0.050
1 2	G40.3 Generalized idiopathic epilepsy and epileptic syndromes		707 685	16.1% 15.6%	0.520	0.014
1 2	G40.4 Other generalized epilepsy and epileptic syndromes		542 507	12.3% 11.5%	0.250	0.025
1 2	G40.A Absence epileptic syndrome		122 110	2.8% 2.5%	0.425	0.017
1 2	G40.B Juvenile myoclonic epilepsy (impulsive petit mal)		106 102	2.4% 2.3%	0.779	0.006
1 2	G40.2 Localization-related (focal) (partial) symptomatic epilepsy and epileptic syndromes with complex partial seizures		746 682	17.0% 15.5%	0.064	0.039
1 2	G40.1 Localization-related (focal) (partial) symptomatic epilepsy and epileptic syndromes with simple partial seizures		495 462	11.3% 10.5%	0.258	0.024
1 2	G40.8 Other epilepsy and recurrent seizures		411 362	9.3% 8.2%	0.065	0.039
1 2	G40.5 Epileptic seizures related to external causes		26 19	0.6% 0.4%	0.295	0.022
1 2	G40.0 Localization-related (focal) (partial) idiopathic epilepsy and epileptic syndromes with seizures of localized onset		244 240	5.6% 5.5%	0.852	0.004
1 2	F30-F39 Mood (affective) disorders		956 908	21.7% 20.7%	0.210	0.027
1 2	F20-F29 Schizophrenia, schizotypal, delusional, and other non-mood psychotic disorders		249 227	5.7% 5.2%	0.300	0.022
1 2	F99-F99 Unspecified mental disorder (F99)		51 49	1.2% 1.1%	0.841	0.004
1 2	X71-X83 Intentional self-harm		23 20	0.5% 0.5%	0.647	0.010
1 2	W00-W19 Slipping, tripping, stumbling and falls		182 166	4.1% 3.8%	0.381	0.019
1 2	T14.91 Suicide attempt		23 23	0.5% 0.5%	1	<0.001
1 2	1013711 Emergency department services		1138 1101	25.9% 25.0%	0.365	0.019
1 2	1013659 Hospital inpatient and observation care services		691 678	15.7% 15.4%	0.702	0.008

SD = standard deviation; std diff. = standardized mean difference.

From 8991 males with epilepsy taking valproate considered for PSM, 2490 (28%) were subsequently withdrawn from treatment and 6501 (72%) remained ON treatment. Table 2 shows their baseline demographic and clinical characteristics after PSM (see Supplementary Table 3 for these characteristics before matching). Supplementary Table 4 shows their co-prescribed ASMs before and after PSM. Outcomes were compared between the 2482 matched males in each cohort.

**Table 2:** Baseline characteristics after propensity score matching: males alone

	2482 withdrawn from valproate (Cohort 1) matched with 2482 remaining ON valproate (Cohort 2)					
Cohort	Characteristic		Patients	% of cohort	*P*-value	Std diff.
1 2	Age at index	26.6 ± 11.3 mean ± SD 26.6 ± 11.2 mean ± SD	2482 2482	100% 100%	0.972	0.001
1 2	White		1641 1643	66.1% 66.2%	0.952	0.002
1 2	Black or African American ethnicity		406 445	16.4% 17.9%	0.142	0.042
1 2	Unknown ethnicity		246 236	9.9% 9.5%	0.632	0.014
1 2	Other ethnicity		123 114	5.0% 4.6%	0.549	0.017
1 2	Asian ethnicity		42 26	1.7% 1.0%	0.051	0.055
1 2	American Indian or Alaska Native ethnicity		17 10	0.7% 0.4%	0.177	0.038
1 2	Native Hawaiian or other Pacific Islander		10 10	0.4% 0.4%	1	<0.001
1 2	R56.9 Unspecified convulsions		916 837	36.9% 33.7%	0.019	0.067
1 2	G40.3 Generalized idiopathic epilepsy and epileptic syndromes		398 368	16.0% 14.8%	0.239	0.033
1 2	G40.4 Other generalized epilepsy and epileptic syndromes		304 284	12.2% 11.4%	0.380	0.025
1 2	G40.A Absence epileptic syndrome		58 46	2.3% 1.9%	0.234	0.034
1 2	G40.B Juvenile myoclonic epilepsy (impulsive petit mal)		54 55	2.2% 2.2%	0.923	0.003
1 2	G40.2 Localization-related (focal) (partial) symptomatic epilepsy and epileptic syndromes with complex partial seizures		450 432	18.1% 17.4%	0.504	0.019
1 2	G40.1 Localization-related (focal) (partial) symptomatic epilepsy and epileptic syndromes with simple partial seizures		302 286	12.2% 11.5%	0.482	0.020
1 2	G40.8 Other epilepsy and recurrent seizures		237 230	9.5% 9.3%	0.734	0.010
1 2	G40.5 Epileptic seizures related to external causes		10 10	0.4% 0.4%	1	<0.001
1 2	G40.0 Localization-related (focal) (partial) idiopathic epilepsy and epileptic syndromes with seizures of localized onset		153 153	6.2% 6.2%	1	<0.001
1 2	F30-F39 Mood (affective) disorders		462 428	18.6% 17.2%	0.208	0.036
1 2	F20-F29 Schizophrenia, schizotypal, delusional, and other non-mood psychotic disorders		127 129	5.1% 5.2%	0.898	0.004
1 2	F99-F99 Unspecified mental disorder (F99)		28 20	1.1% 0.8%	0.246	0.033
1 2	X71-X83 Intentional self-harm		12 10	0.5% 0.4%	0.669	0.012
1 2	W00-W19 Slipping, tripping, stumbling and falls		97 87	3.9% 3.5%	0.452	0.021
1 2	T14.91 Suicide attempt		11 10	0.4% 0.4%	0.827	0.006
1 2	1013711 Emergency department services		619 619	24.9% 24.9%	1	<0.001
1 2	1013659 Hospital inpatient and observation care services		388 369	15.6% 14.9%	0.453	0.021

SD = standard deviation; std diff. = standardized mean difference.

From 5243 females with epilepsy taking valproate considered for PSM, 1880 (36%) were subsequently withdrawn from treatment and 3363 (64%) remained ON treatment. Table 3 shows their baseline demographic and clinical characteristics after PSM (see Supplementary Table 5 for these characteristics before matching). Supplementary Table 6 shows their co-prescribed ASMs before and after PSM, respectively. Outcomes were compared between the 1812 matched females in each cohort. One hundred and seven of the females taking valproate subsequently became pregnant during recruitment. Sixty-three per cent (68 females) were withdrawn from treatment and 36% (39 females) remained on treatment. The PSM process failed to fully match these two subgroups of females due to the small numbers, meaning valid comparisons could not be made.

**Table 3:** Baseline characteristics after propensity score matching: females alone

	1812 withdrawn from valproate (Cohort 1) matched with 1812 remaining ON valproate (Cohort 2)					
Cohort	Characteristic		Patients	% of cohort	*P*-value	Std diff.
1 2	Age at index	28.4 ± 11.8 mean ± SD 28.5 ± 11.8 mean ± SD	1812 1812	100% 100%	0.665	0.014
1 2	White		1207 1206	66.6% 66.6%	0.972	0.001
1 2	Black or African American ethnicity		281 297	15.5% 16.4%	0.468	0.024
1 2	Unknown ethnicity		189 191	10.4% 10.5%	0.914	0.004
1 2	Other ethnicity		80 72	4.4% 4.0%	0.507	0.022
1 2	Asian ethnicity		41 36	2.3% 2.0%	0.565	0.019
1 2	American Indian or Alaska Native ethnicity		12 10	0.7% 0.6%	0.669	0.014
1 2	Native Hawaiian or other Pacific Islander		10 0	0.6% 0%	0.002	0.105
1 2	R56.9 Unspecified convulsions		684 653	37.7% 36.0%	0.286	0.035
1 2	G40.3 Generalized idiopathic epilepsy and epileptic syndromes		285 269	15.7% 14.8%	0.460	0.025
1 2	G40.4 Other generalized epilepsy and epileptic syndromes		221 212	12.2% 11.7%	0.645	0.015
1 2	G40.A Absence epileptic syndrome		61 52	3.4% 2.9%	0.390	0.029
1 2	G40.B Juvenile myoclonic epilepsy (impulsive petit mal)		47 37	2.6% 2.0%	0.270	0.037
1 2	G40.2 Localization-related (focal) (partial) symptomatic epilepsy and epileptic syndromes with complex partial seizures		273 248	15.1% 13.7%	0.237	0.039
1 2	G40.1 Localization-related (focal) (partial) symptomatic epilepsy and epileptic syndromes with simple partial seizures		183 168	10.1% 9.3%	0.400	0.028
1 2	G40.8 Other epilepsy and recurrent seizures		169 156	9.3% 8.6%	0.450	0.025
1 2	G40.5 Epileptic seizures related to external causes		15 16	0.8% 0.9%	0.857	0.006
1 2	G40.0 Localization-related (focal) (partial) idiopathic epilepsy and epileptic syndromes with seizures of localized onset		85 77	4.7% 4.2%	0.520	0.021
1 2	F30-F39 Mood (affective) disorders		462 463	25.5% 25.6%	0.970	0.001
1 2	F20-F29 Schizophrenia, schizotypal, delusional, and other non-mood psychotic disorders		112 114	6.2% 6.3%	0.891	0.005
1 2	F99-F99 Unspecified mental disorder (F99)		23 24	1.3% 1.3%	0.883	0.005
1 2	X71-X83 Intentional self-harm		12 10	0.7% 0.6%	0.669	0.014
1 2	W00-W19 Slipping, tripping, stumbling and falls		75 76	4.1% 4.2%	0.934	0.003
1 2	T14.91 Suicide attempt		11 12	0.6% 0.7%	0.834	0.007
1 2	1013711 Emergency department services		489 480	27.0% 26.5%	0.736	0.011
1 2	1013659 Hospital inpatient and observation care services		277 264	15.3% 14.6%	0.545	0.020

SD = standard deviation; std diff. = standardized mean difference.

### All-cause deaths

**Figure 1:**
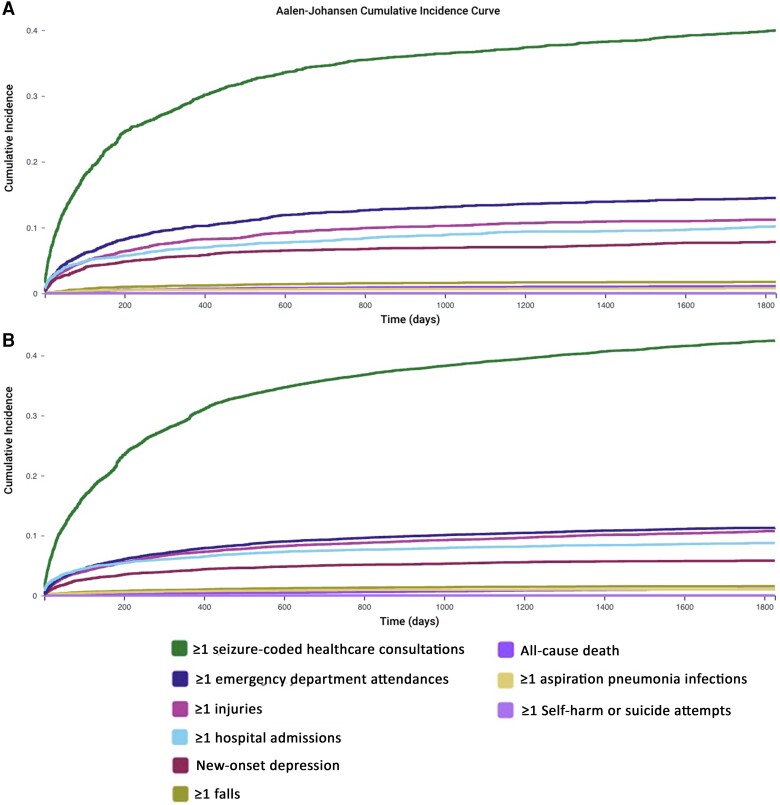
**Cumulative incidence panel of results from males and females combined**. (**A**) Those withdrawn from valproate. (**B**) Those who remained ON valproate.

Sixteen more people died in the group of males and females withdrawn from valproate, 1% higher than in the group remaining ON valproate (Table 4). However, there was no evidence to suggest that this change was more likely to occur than chance (HR 1.109, CI 0.927–1.327). When looked at separately, mortality was not increased for males (HR 1.117, CI 0.879–1.419) or females (HR 1.013, CI 0.769–1.335) withdrawn from valproate (Table 4). The cumulative incidence for death was higher than for aspiration pneumonia and higher than for self-harm or suicide, suggesting these outcomes may have been outcompeted by death (Fig. 1 and Supplementary Table 7).

**Table:** Table 4 Survival analysis and event frequency results

	Cohort statistics				
Outcome name	Valproate treatment	No. in cohort	No. with outcome (% of cohort)	Mean event frequency ± SD	HR (95% CI)
**Males and females combined**					
All-cause death	Withdrawn Continued	4396 4396	248 (6%) 232 (5%)	n/a n/a	1.109 (0.927–1.327)
≥1 emergency department attendances	Withdrawn Continued	4396 4396	1971 (45%) 1733 (39%)	4 ± 11 4 ± 15	1.236 (1.159–1.319)*
≥1 hospital admissions	Withdrawn Continued	4396 4396	1610 (37%) 1460 (33%)	3 ± 10 2 ± 9	1.160 (1.081–1.246)*
≥1 seizure-coded healthcare consultations	Withdrawn Continued	4396 4396	2491 (57%) 2492 (57%)	5 ± 12 5 ± 14	1.048 (0.991–1.108)
≥1 falls	Withdrawn Continued	4396 4396	524 (12%) 464 (11%)	0 ± 1 0 ± 1	1.179 (1.041–1.336)*
≥1 injuries	Withdrawn Continued	4396 4396	1601 (36%) 1537 (35%)	2 ± 8 2 ± 9	1.095 (1.021–1.174)*
≥1 burns	Withdrawn Continued	4396 4396	66 (2%) 43 (1%)	0 ± 1 0 ± 0	1.592 (1.084–2.337)*
≥1 aspiration pneumonia infections	Withdrawn Continued	4396 4396	189 (4%) 244 (6%)	0 ± 1 0 ± 2	0.793 (0.656–0.959)*
New-onset depression	Withdrawn Continued	3155 3376	296 (9%) 253 (8%)	n/a n/a	1.323 (1.119–1.565)*
≥1 Self-harm or suicide attempts	Withdrawn Continued	4396 4396	85 (2%) 81 (2%)	0 ± 1 0 ± 1	1.085 (0.800–1.470)
**Males alone**					
All-cause death	Withdrawn Continued	2482 2482	139 (6%) 129 (5%)	n/a n/a	1.117 (0.879–1.419)
≥1 emergency department attendances	Withdrawn Continued	2482 2482	1077 (43%) 974 (39%)	3 ± 10 3 ± 14	1.181 (1.083–1.288)*
≥1 hospital admissions	Withdrawn Continued	2482 2482	835 (34%) 767 (31%)	2 ± 8 2 ± 9	1.132 (1.027–1.249)*
≥1 seizure-coded healthcare consultations	Withdrawn Continued	2482 2482	1377 (55%) 1386 (56%)	4 ± 11 4 ± 10	1.032 (0.958–1.112)
≥1 falls	Withdrawn Continued	2482 2482	281 (11%) 227 (9%)	0 ± 1 0 ± 1	1.298 (1.090–1.546)*
≥1 injuries	Withdrawn Continued	2482 2482	927 (37%) 871 (35%)	2 ± 8 2 ± 8	1.129 (1.029–1.239)*
≥1 burns	Withdrawn Continued	2482 2482	30 (1%) 20 (1%)	0 ± 0 0 ± 0	1.551 (0.881–2.730)
≥1 aspiration pneumonia infections	Withdrawn Continued	2482 2482	117 (5%) 121 (5%)	0 ± 1 0 ± 2	0.994 (0.771–1.282)
New-onset depression	Withdrawn Continued	1882 2011	137 (7%) 144 (7%)	n/a n/a	1.057 (0.837–1.336)
≥1 Self-harm or suicide attempts	Withdrawn Continued	2482 2482	42 (2%) 43 (2%)	0 ± 2 0 ± 0	1.008 (0.659–1.543)
**Females alone**					
All-cause death	Withdrawn Continued	1812 1812	100 (6%) 102 (6%)	n/a n/a	1.013 (0.769–1.335)
≥1 emergency department attendances	Withdrawn Continued	1812 1812	844 (47%) 740 (41%)	4 ± 12 4 ± 15	1.242 (1.125–1.371)*
≥1 hospital admissions	Withdrawn Continued	1812 1812	730 (40%) 670 (37%)	3 ± 12 3 ± 10	1.147 (1.033–1.274)*
≥1 seizure-coded healthcare consultations	Withdrawn Continued	1812 1812	1045 (58%) 1081 (60%)	5 ± 13 5 ± 17	1.006 (0.924–1.095)
≥1 falls	Withdrawn Continued	1812 1812	228 (13%) 204 (11%)	0 ± 1 0 ± 1	1.159 (0.959–1.400)
≥1 injuries	Withdrawn Continued	1812 1812	632 (35%) 634 (35%)	3 ± 10 2 ± 11	1.033 (0.925–1.154)
≥1 burns	Withdrawn Continued	1812 1812	35 (2%) 22 (1%)	0 ± 1 0 ± 0	1.644 (0.965–2.803)
≥1 aspiration pneumonia infections	Withdrawn Continued	1812 1812	62 (3%) 93 (5%)	0 ± 1 0 ± 1	0.682 (0.494–0.940)*
New-onset depression	Withdrawn Continued	1188 1308	151 (13%) 128 (10%)	n/a n/a	1.359 (1.074–1.720)*
≥1 Self-harm or suicide attempts	Withdrawn Continued	1812 1812	40 (2%) 39 (2%)	0 ± 1 0 ± 1	1.055 (0.679–1.640)

Where the sum of the male only + female only numerators and denominators add up to different totals than in the male and female combined analysis, this is due to propensity score matching (PSM) differences. The PSM process is necessarily rerun on each of these analysis groups separately, and the same matches are not necessarily appropriate for each independent run when trying to maintain adequate matching overall in the group. Matching in the male and female combined analysis also takes account of the 175 people who were of unknown gender. CI = confidence interval; HR = hazard ratio; n/a = not applicable; SD = standard deviation.

^*^Statistically significant result.

### All-cause emergency department attendance

**Figure 2:**
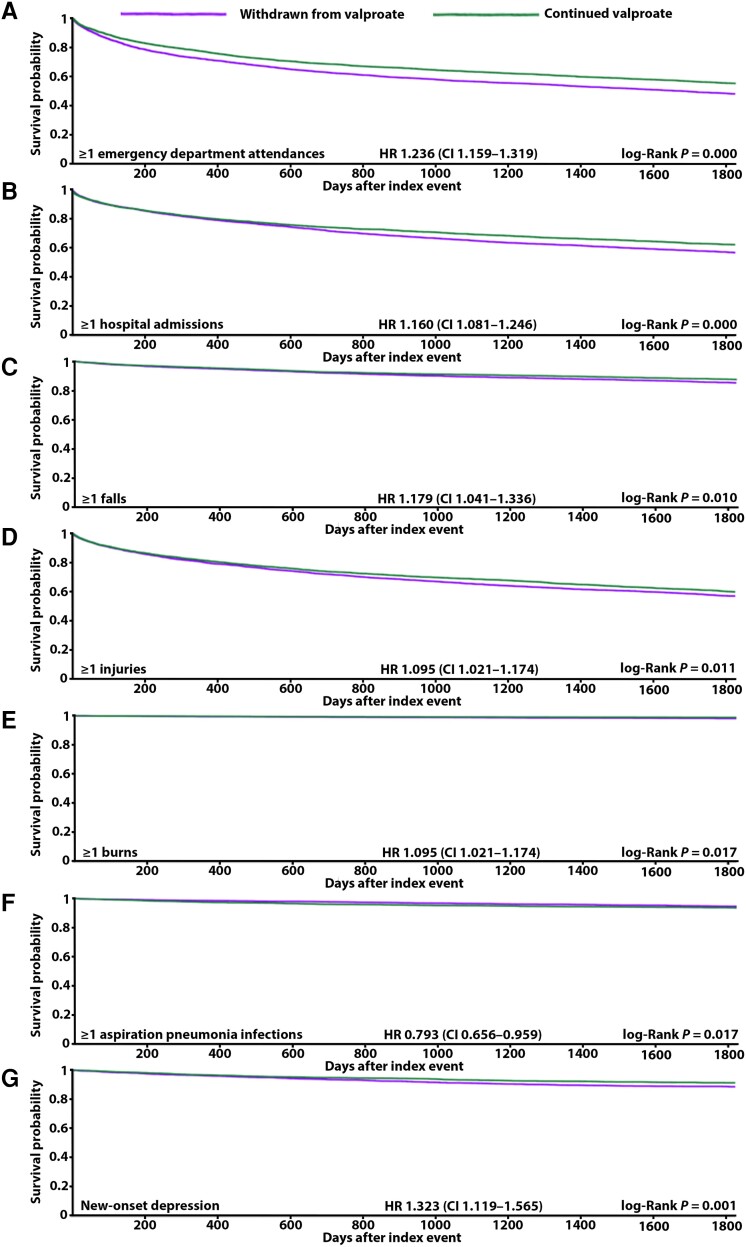
**Kaplan-Meier panel of significant results from males and females combined**. (**A**) Emergency department attendances. (**B**) Hospital admissions. (**C**) Falls. (**D**) Injuries. (**E**) Burns. (**F**) Aspiration pneumonia. (**G**) New-onset depression. CI = confidence interval; HR = hazard ratio.

**Figure 3:**
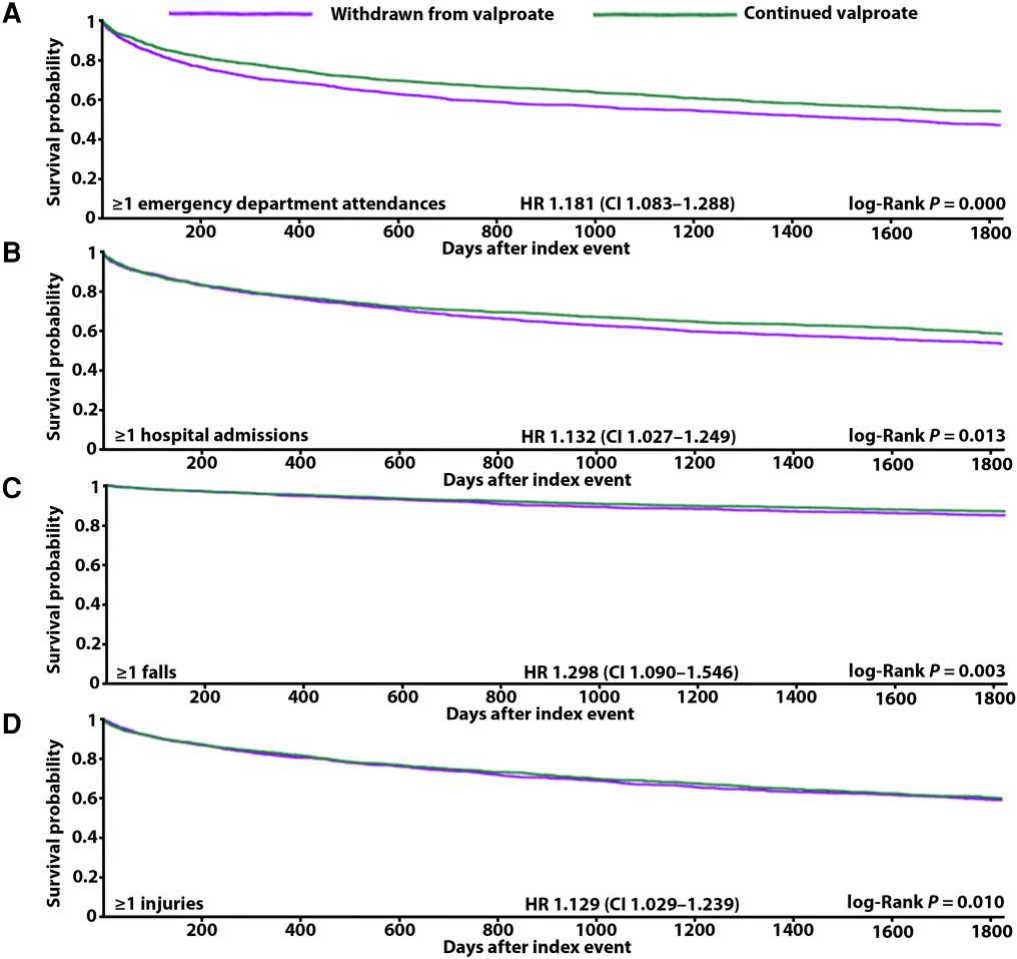
**Kaplan-Meier panel of significant results from males alone**. (**A**) Emergency department attendances. (**B**) Hospital admissions. (**C**) Falls. (**D**) Injuries. CI = confidence interval; HR = hazard ratio.

**Figure 4:**
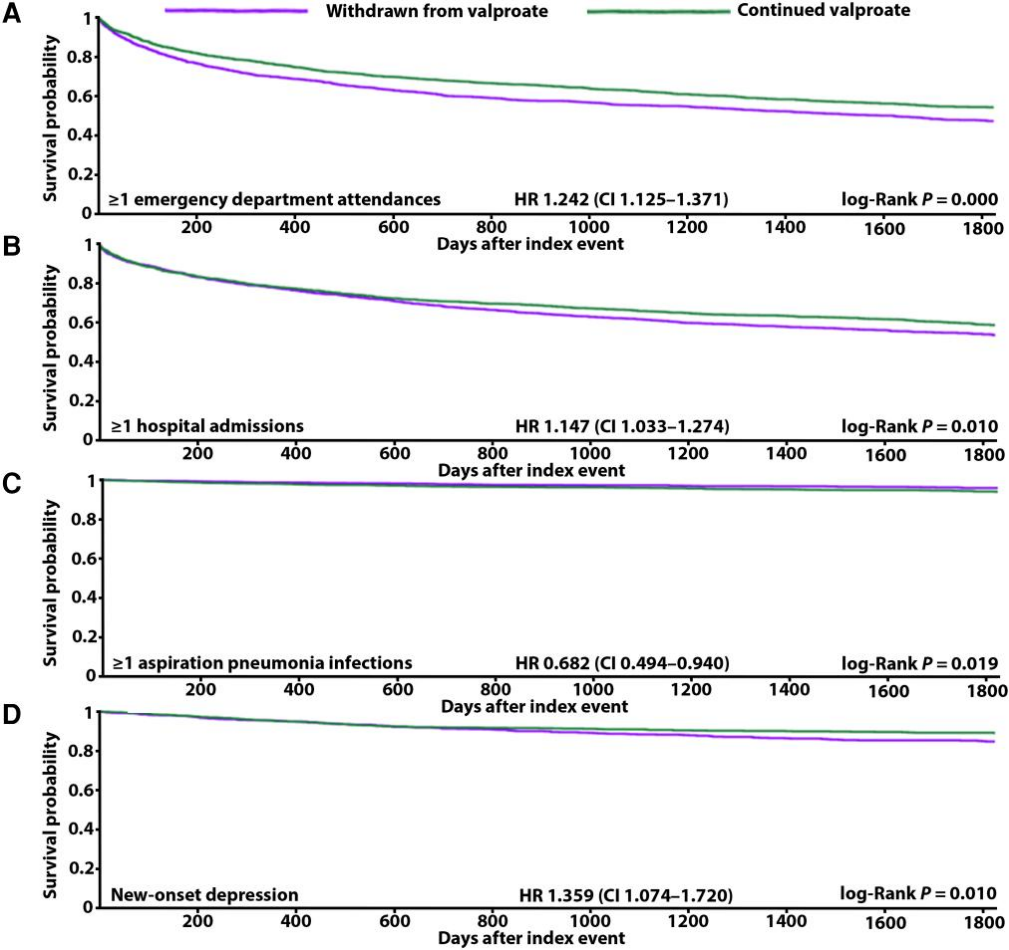
**Kaplan-Meier panel of significant results from females alone**. (**A**) Emergency department attendances. (**B**) Hospital admissions. (**C**) Aspiration pneumonia. (**D**) New-onset depression. CI = confidence interval; HR = hazard ratio.

Whether analysed together or separately (Table 4), males and females withdrawn from valproate were at significantly increased risk of experiencing one or more emergency department attendances over 5 years [overall HR 1.236, CI 1.159–1.319 (Fig. 2A), male HR 1.181, CI 1.083–1.288 (Fig. 3A), female HR 1.242, CI 1.125–1.371 (Fig. 4A)]. In the group of males and females combined, 45% and 39% of those withdrawn from and remaining ON valproate, respectively, experienced this outcome. This 5% increase in risk was resultant from 238 more attending emergency department in the group withdrawn from valproate. A 4% and 6% increase in risk was seen specifically in males and females, respectively, when their analysis was separated out. Overall, the people who did experience emergency department attendance had an average of four of these over 5 years whether withdrawn from or remaining ON valproate.

### All-cause hospital admission

Whether analysed together or separately (Table 4), males and females withdrawn from valproate were at significantly increased risk of experiencing one or more hospital admissions over 5 years [overall HR 1.160, CI 1.081–1.246 (Fig. 2B), male HR 1.132, CI 1.027–1.249 (Fig. 3B), female HR 1.147, CI 1.033–1.274 (Fig. 4B)]. In the group of males and females combined, 37% and 33% of those withdrawn from and remaining ON valproate, respectively, experienced this outcome. This 4% increase in risk resulted from 150 more being admitted to hospital in the group withdrawn from valproate. A 3% increase in risk was seen similarly for males and for females when their analysis was separated out. Overall, the people who did experience hospital admission had an average of three over 5 years and two over 5 years in the group withdrawn from and remaining ON valproate, respectively.

The composite measure of emergency department attendance and hospital admission tested to account for potentially competing risk between these two outcomes retained similar trends to those observed for each outcome in isolation: whether analysed together or separately, males and females withdrawn from valproate were at significantly increased risk of experiencing one or more emergency department attendances and/or hospital admissions over 5 years (overall HR 1.244, CI 1.174–1.318, male HR 1.167, CI 1.079–1.262, female HR 1.260, CI 1.155–1.375). This composite outcome affected 56% and 49% of the people withdrawn from and remaining ON valproate, respectively. This 7% increase in risk was resultant from 285 more experiencing the outcome in the group withdrawn from valproate. A 5% and 7% increase in risk was seen specifically in males and females, respectively, when their analysis was separated out. There was an average of six events occurring in each group over the 5-year follow-up.

### Seizure-coded healthcare consultations

For males and females, whether analysed together or separately (Table 4), a similar risk for one or more seizure-coded healthcare consultations over 5 years was seen whether withdrawn from or remaining ON valproate (overall HR 1.048, CI 0.991–1.108, male HR 1.032, CI 0.958–1.112, female HR 1.006, CI 0.924–1.095). Fifty-seven per cent of both cohorts experienced this outcome (2491 of those withdrawn from valproate and 2492 of those remaining ON valproate). Overall, the 57% who did experience one or more seizure-coded healthcare consultations had an average of five over the 5 years in both groups.

### Falls

When analysed together (Table 4), males and females withdrawn from valproate were at significantly increased risk of one or more falls over 5 years (HR 1.179, CI 1.041–1.336) (Fig. 2C). Twelve per cent and 11% of those withdrawn from and remaining ON valproate experienced this outcome, respectively. This 1% increase in risk was resultant from 60 more people falling in the group withdrawn from valproate. When looked at separately (Table 4), the significantly increased risk of falls remained in males but not females [male HR 1.298, CI 1.090–1.546 (Fig. 3C), female HR 1.159, CI 0.959–1.400]. The 54 more males falling in the group withdrawn from valproate corresponded to a significant 2.2% increase in risk, whereas the 24 more females falling in the group withdrawn from valproate corresponded to a lesser 1.3% increase in risk (not significant). Overall, the absolute frequency of falls was low, with a mean figure of 0 in both groups over 5 years.

### Injuries

When analysed together (Table 4), males and females withdrawn from valproate were at significantly increased risk of one or more injuries over 5 years (HR 1.095, CI 1.021–1.174) (Fig. 2D). Thirty-six per cent and 35% of those withdrawn from and remaining ON valproate experienced this outcome, respectively. This 1% increase in risk was resultant from 64 more people being injured in the group withdrawn from valproate. When looked at separately (Table 4), the significantly increased risk of injuries remained in males but not females [male HR 1.129, CI 1.029–1.239 (Fig. 3D), female HR 1.033, CI 0.925–1.154]. The 56 more males injured in the group withdrawn from valproate corresponded to a significant 2% increase in risk, whereas approximately equal numbers of females were injured: 632 and 634 in those withdrawn from and remaining ON valproate, respectively. Overall, the people who did experience injuries had an average of two over the 5 years in both groups.

### Burns

When analysed together (Table 4), males and females withdrawn from valproate were at significantly increased risk of one or more burn incidents over 5 years (HR 1.592, CI 1.084–2.337) (Fig. 2E). Two per cent and 1% of those withdrawn from and remaining ON valproate experienced this outcome, respectively. This 1% increase in risk was resultant from 23 more people being burnt in the group withdrawn from valproate. When looked at separately (Table 4), the 10 more males and 13 more females experiencing these burns in the group withdrawn from valproate did not reach significance (male HR 1.551, CI 0.881–2.730, female HR 1.644, CI 0.965–2.803). Overall, the absolute frequency of burn incidents was low, with a mean figure of 0 in both groups over 5 years.

### Aspiration pneumonia

When analysed together (Table 4), males and females withdrawn from valproate appeared to be at significantly reduced risk of one or more aspiration pneumonia infections over 5 years (HR 0.793, CI 0.656–0.959) (Fig. 2F). Four per cent and 6% of those withdrawn from and remaining ON valproate experienced this outcome. This 2% decrease in risk was resultant from 55 fewer people developing aspiration pneumonia in the group withdrawn from valproate. However, death may have inferred a substantial competitive risk against this outcome given its higher cumulative incidence over aspiration pneumonia (Fig. 1). This competitive risk was more evident in those withdrawn from valproate than in those remaining ON valproate. In those withdrawn from valproate, the difference in cumulative incidence between death (cumulative incidence 0.0109) and aspiration pneumonia (cumulative incidence 0.0077) was 0.0032. Less of a difference than this was seen in those remaining ON valproate, where the difference in cumulative incidence between death (cumulative incidence 0.0104) and aspiration pneumonia (cumulative incidence 0.0102) was 0.0002. When looked at separately (Table 4), the apparent reduction in risk of aspiration pneumonia remained in females but not males [female HR 0.682, CI 0.494–0.940 (Fig. 4C), male HR 0.994, CI 0.771–1.282]. Overall, the absolute frequency of aspiration pneumonia infections was low, with a mean figure of 0 in both groups over 5 years.

### New-onset depression

In the group of males and females combined, 1241 and 1020 of those withdrawn from and remaining ON valproate, respectively, were excluded from results because they already had depression coded prior to our assessment period. For those remaining in the cohorts, when analysed together (Table 4), males and females withdrawn from valproate were at significantly increased risk of developing new-onset depression over 5 years (HR 1.323, CI 1.119–1.565) (Fig. 2G). Nine per cent and 8% of those withdrawn from and remaining ON valproate experienced this outcome, respectively. This 1% increase in risk was resultant from 43 more people developing depression in the group withdrawn from valproate. When looked at separately (Table 4), the significantly increased risk of new-onset depression remained in females but not males [female HR 1.359, CI 1.074–1.720 (Fig. 4D), male HR 1.057, CI 0.837–1.336].

### Self-harm or suicide attempts

There was no evidence to suggest an increased risk of self-harm or suicide attempts in PWE withdrawn from valproate (overall HR 1.085, CI 0.800–1.470, male HR 1.008, CI 0.659–1.543, female HR 1.055, CI 0.679–1.640, Table 4). A consistent proportion of 2% experienced one or more self-harm or suicide attempts in both cohorts. Overall, the absolute frequency of self-harm or suicide attempts was low, with a mean figure of 0 in both groups over 5 years.

## Discussion

To our knowledge, this is the largest study assessing the risks of valproate withdrawal in PWE. Results are largely drawn from a US population, but other countries are also represented, mainly in Europe. We show that PWE withdrawn from valproate are at increased risk of experiencing one or more emergency department attendances, hospital admissions, falls, injuries, burns and new-onset depression. Of these, risks of emergency department attendance and/or hospital admission are highest: ∼7% more in those withdrawn from valproate than in those remaining ON valproate. For the remaining risks (falls, injuries, burns, new-onset depression), the risks are 1%–2% higher. The risks seen between males and females are generally similar for emergency department attendance and/or hospital admission. Falls and injuries seem to be more common in males, whilst new-onset depression seems to be more common for females. This is the first time these wider morbidity outcomes have been assessed in PWE within a context of valproate withdrawal, and the findings may help facilitate a broader conversation about the potential risks and benefits of this between clinicians and patients,^31,32^ understanding that there is more to discuss about epilepsy than seizures alone.

The data do not show a difference in one or more seizure-coded healthcare consultations between those withdrawn from versus remaining ON valproate. However, in cohorts of PWE, it would be unlikely that an increased signal for emergency department attendances, hospital admissions, falls, injuries and burns would be unrelated to increased seizures. Therefore, it is possible a number of breakthrough seizures were not captured by healthcare coding. Whilst there are a substantial number of studies validating the accuracy of seizure symptom codes in capturing epilepsy as a disease (when paired with co-prescribed ASMs),^40^ few studies have been able to interrogate whether seizure symptom coding, as a process, can actually pick up all of the seizures a patient experiences. Our clinical experience would suggest this is unlikely for a number of reasons: patients do not always know they have had seizures, nor are all their known seizures severe enough to come to the attention of healthcare providers, and not all of the seizures brought to the attention of healthcare provides (e.g. in a seizure diary) are then routinely extracted and coded in a 1:1 ratio with seizure symptom codes. Identifying such granular detail would require hand-reviewing medical records. When this has been done previously, it has usually shown worsening seizure control when valproate is withdrawn, regardless of whether or not switched to another ASM.^18,23,24,26,27^

There is no evidence to support a significantly increased risk of self-harm/suicide attempts in PWE withdrawn from valproate in these data. A signal is seen for fewer aspiration pneumonia infections in those withdrawn from valproate. However, this is interpreted with caution as it is also seen that death may have inferred a substantial competitive risk against this outcome, particularly in those withdrawn from valproate. Overall, however, the data show no evidence to support a significantly increased risk of death in PWE withdrawn from valproate. This finding should be interpreted with knowledge of the sourcing structure for mortality data in TriNetX. The main source for mortality data in TriNetX is electronic health records (EHRs), capturing >4 million provider-supplied deaths. As data sourced from EHRs alone may underestimate deaths due to some occurring outside of healthcare information workstreams, TriNetX source additional mortality data from outside EHRs, including from government (Social Security Administration’s Master Death File, covering 20% of US deaths), private obituaries (covering 40% of US deaths) and private claims (covering 20% of US deaths).^39^ TriNetX report that their mortality data cover ∼85% of in the US population through these linked strategies.^37^ Therefore, our study is likely to have captured the vast majority of deaths and sampled the trends adequately. However, an underestimation of the overall number of deaths remains possible, which would influence the competitive risk potential over other outcomes. Further studies outside of the TriNetX network are needed to help validate if the lack of excess mortality seen here with valproate withdrawal is reproducible. A recent study of national valproate prescribing data from Clinical Practice Research Datalink (CPRD, which encompasses electronic health data from 60 million patients in the UK)^64^ appears to also reproduce the lack of excess mortality we observed with valproate withdrawal, although the study remains in preprint currently.^30^

It is important that doctors and patients can have a fully informed discussion about safety when considering valproate withdrawal. This study helps facilitate that by providing several as yet unquantified risk estimates of morbidity and mortality associated with valproate withdrawal. We have ensured the outcomes we selected to reflect morbidity and mortality are meaningful to people affected by epilepsy through a public engagement process. We include the first dedicated large-sample assessment of valproate withdrawal in males alone. This may be helpful in the context of the forthcoming regulatory changes scheduled to limit valproate prescribing for males.^8^ The findings also help inform similar discussions between females with epilepsy and their clinicians, using data from a much larger study than any previously reported of this topic in females, assessing a broader range of withdrawal risk outcomes beyond seizures alone.^17,18,21-29^

Patients invited to withdraw from valproate need to make judgements on the maximum acceptable risk of harm (MAR) for an expected health benefit. Discrete choice experiments have helped inform this area previously.^65^ These have shown that reduction in seizure frequency is unanimously the most important treatment outcome (benefit) in PWE.^65^ However, PWE also prioritize reduction in the risk of adverse effects, and this differs by patient subgroup. Specifically, females of childbearing potential with epilepsy tend to be willing to sacrifice a 5% reduction in the probability of 12-month seizure remission to reduce the risk of foetal abnormality by 1%.^65^ The decisions males will make in this scenario are not yet clear. Further research is needed. Our findings may help inform the MAR calculations for males in such studies.

### Study strengths

We report the largest study of valproate withdrawal to date,^17,18,21-29^ drawing data from young adults in multiple countries around the world to allow more generalizable conclusions to be made from real-world data taken directly from clinical practice in real-time. This means that not only will our results reflect trends following valproate withdrawal in remission, but also situations where valproate was withdrawn for safety concerns, adverse effects, patient preference and even in relation to the relevant national regulatory changes in each country. Such a large study size has also allowed us to look at multiple outcomes that are likely to be meaningful to PWE and have not yet been assessed following valproate withdrawal previously because of limited study sizes.^17,18,21-29^

### Limitations

This study should be interpreted within the context of its limitations. First, as for all observational studies, inferences can be drawn about associations alone, not causality. However, we do acknowledge that the novel method of target trial emulation does allow for some confidence in drawing causal inferences from observational data.^66^ Such a design was not possible for this study as g-methods, such as inverse probability weighting, are not currently available in the TriNetX query builder format we used. A target trial emulation would be the logical next line of enquiry for future studies, using raw data extracts from TriNetX or other large healthcare datasets like Clinical Practice Research Datalink (CPRD), and this is work we have commenced recently.^67^ It is intuitive that the risks associated with valproate withdrawal we identified in the current study could also be associated with withdrawal from other ASMs. However, prescribing these other ASMs is not being restricted in the same manner as valproate,^8,14^ meaning an assessment of outcomes following valproate withdrawal in and of itself remains directly relevant to the questions clinicians already face from females ON valproate and will face from males ON valproate. Direct head-to-head comparisons of outcomes between withdrawal of valproate and withdrawal of other ASMs was not possible using the TriNetX query builder, but will be helpful in future. It will also be helpful to compare morbidity and mortality between different ASMs switched to from valproate to understand which alternatives are safest and most effective.^67^

The signal for worsening morbidity OFF valproate seen could be us simply capturing withdrawal from valproate as a marker of severe epilepsy with a poor prognosis needing escalation of treatment. However, we reduced the chances of this being the case by ‘levelling off’ these morbidities against one another in the year prior to commencing comparisons through matching cohorts by type of epilepsy, the occurrence of one or more seizure-coded healthcare consultations, emergency department attendances, hospital admissions, psychiatric comorbidities, falls and self-harm/suicide attempts. It remains possible we could have overestimated incident depression if participants were coded before the available electronic health record (particularly before 2008). However, we wouldn’t expect this factor to affect the comparison groups differently from one another.

Owing to limitations of using the TriNetX query builder, we were unable to stratify certain outcomes by whether or not they were epilepsy-related, meaning the signal seen may have been compounded or diluted by those unrelated to epilepsy. We were also unable to use weighting schemes to correct for data clustering within HCOs. We were unable to match cohorts by country or type of HCO, which could have confounded results. However, the predominance of large academic HCOs in TriNetX and over-representation from the USA are likely to have limited the impact of this confounder. For privacy reasons, the disclosure of HCO-specific data is not permitted by TriNetX.^37^ The possibility of immortal time biases also could not be excluded as the censorship occurring after the last fact did not take into account prior intervals during which participants were temporarily deregistered from HCOs in the TriNetX catchment. Fill data were also unavailable, making it more difficult to be certain of whether participants prescribed valproate fully adhered to the medication. However, even studies with fill data are challenged because medication fills do not necessarily confirm that patients have subsequently taken their medication. The best marker for adherence is plasma drug levels, although not every patient ON valproate needs to have these measured. In a *post hoc* validation exercise, we were able to demonstrate that plasma valproate levels were detectable at the last reading taken during the 5-year follow-up of 97.4% of participants remaining ON valproate who fulfilled our study inclusion criteria and who had also had serum valproate levels checked (Supplementary Fig. 1). This would suggest reasonable medication adherence.

## Conclusions

Males and females with epilepsy taking valproate should be adequately counselled about not only the potential risks to fertility and increased teratogenicity if they stay ON valproate, but also any potential harms associated with coming OFF valproate. Based on our work, the potential harms could consist of increased emergency department attendances, hospital admissions, falls, injuries, burns and new-onset depression. Further work externally is required to fully understand whether or not mortality increases in association with valproate withdrawal as the absolute numbers we found were slightly higher in the group withdrawn from valproate, but not significantly so. However, it has been suggested that TriNeX may underestimate deaths.^38^ Furthermore, we reported all-cause deaths here as that is what we had available in TriNetX. However, it will be important to investigate epilepsy-related deaths, specifically, in future, as we have planned.^67^ This is because a signal for increased epilepsy-related deaths can be missed (diluted) when they are measured together with the remaining all-cause deaths.^46,47^ Overall, this study provides secular data and trends that may help inform the decision-making process surrounding valproate withdrawal in young males and females.

## Supplementary Material

awae128_Supplementary_Data

## Data Availability

The TriNetX system returned aggregate results of the selected analyses within their online query builder. The aggregate results are available at: https://drive.google.com/drive/folders/1DfupZPAdM2HJPRcgCNnRUrWsnsbqlqLJ?usp=sharing. Access to raw data generating the aggregate results would require an approved user request to TriNetX (www.trinetx.com/about-trinetx/contact). This would allow other researchers to identify similar cohorts of participants as were used in our analysis; however, TriNetX is a live platform with new data being added from participating HCOs in real-time, meaning exact counts will vary. For the purpose of open access, the author has applied a Creative Commons Attribution (CC BY) license to any Author Accepted Manuscript version arising.
