# Supplementary material for: Morbidity and mortality risks associated with valproate withdrawal in young adults with epilepsy
Source: Brain. 2024 Apr 24;147(10):3426–41. doi: 10.1093/brain/awae128 (PMC11449131; doi:10.1093/brain/awae128)
Supplement: awae128_Supplementary_Data [file awae128_supplementary_data.zip › brain-2023-02344-File010.pdf]

Supplementary Fig. 1 Bar graph summarising plasma valproate levels when last checked in men and women remaining on valproate.

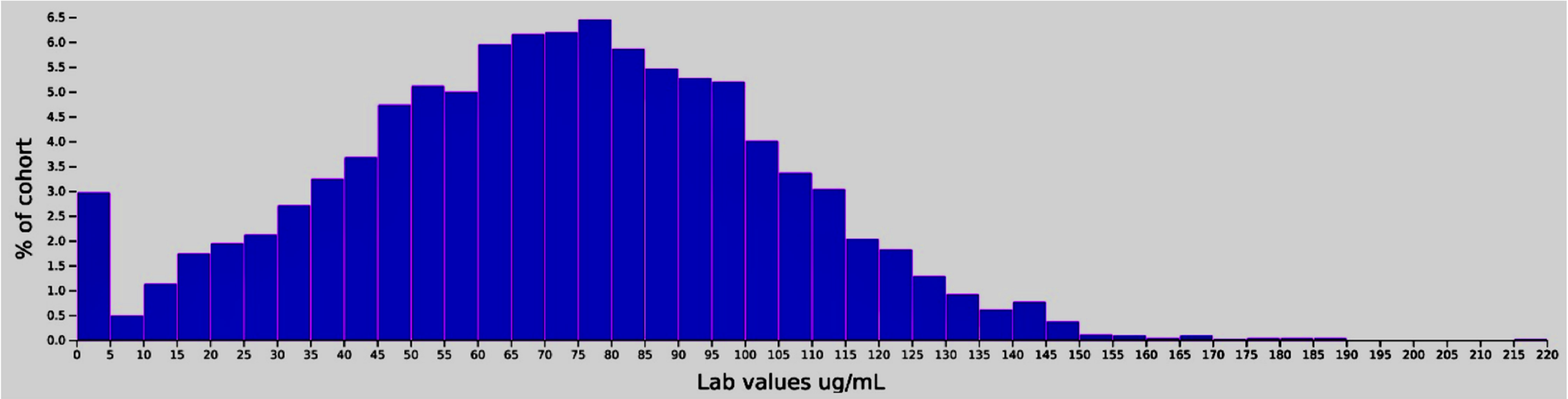

Cohort statistics:

Patients in cohort = 10,063,

Patients with plasma valproate levels measured during follow-up = 5,768

Mean valproate level when last checked ± SD = 71 ± 32 ug/mL
