# Supplementary material for: Morbidity and mortality risks associated with valproate withdrawal in young adults with epilepsy
Source: Brain. 2024 Apr 24;147(10):3426–41. doi: 10.1093/brain/awae128 (PMC11449131; doi:10.1093/brain/awae128)
Supplement: awae128_Supplementary_Data [file awae128_supplementary_data.zip › brain-2023-02344-File011.pdf]

Supplementary material

Supplement S1: ICD-10-CM codes used for injuries 2

Supplement S2: Antiseizure medications used for propensity score matching 3

Table S1: Baseline characteristics before propensity score matching – men and women combined 4

Table S2: Co-prescribed antiseizure medications before and after propensity score matching – men and women combined 5

Table S3: Baseline characteristics before propensity score matching – men alone 6

Table S4: Co-prescribed antiseizure medications before and after propensity score matching – men alone 7

Table S5: Baseline characteristics before propensity score matching – women alone 8

Table S6: Co-prescribed antiseizure medications before and after propensity score matching – women alone 9

Table S7: Competing Risks Results Table – men and women combined 10

Supplement S1: ICD-10-CM codes used for injuries

ICD-10-CM codes used for Injuries

- Injuries to the head (S00-S09)
- Injuries to the wrist, hand and fingers (S60-S69)
- Injuries to the knee and lower leg (S80-S89)
- Injuries to the ankle and foot (S90-S99) Injuries to the abdomen, lower back, lumbar spine, pelvis and external genitals (S30-S39)
- Injuries to the shoulder and upper arm (S40-S49)
- Injuries to the thorax (S20-S29)
- Injuries to the elbow and forearm (S50-S59)
- Injuries to the neck (S10-S19)
- Injuries to the hip and thigh (S70-S79)
- Injuries involving multiple body regions (T07)
- Injury of unspecified body region (T14)

Supplement S2: Antiseizure medications used for propensity score matching

RxNorm

Brivaracetam

Cannabidiol

Carbamazepine

Clobazam

Clonazepam

Diazepam

Eslicarbazepine

Ethosuximide

Felbamate

Fosphenytoin

Gabapentin

Lacosamide

Lamotrigine

Levetiracetam

Lorazepam

Methsuximide

Midazolam

Oxcarbazepine

Paramethadione

Perampanel

Phenobarbital

Phenytoin

Primidone

Rufinamide

Tiagabine

Topiramate

Valproate

Vigabatrin

Zonisamide

Table S1: Baseline characteristics before propensity score matching – men and women combined

	4436 withdrawn from valproate (cohort 1) and 9976 remaining on valproate (cohort 2)				
**Cohort**	**Characteristic**	**Patients**	**% of Cohort**	**P-Value**	**Std diff.**
1<br>2	Age at index 27.5 +/- 11.5 Mean ± SD<br>27.2 +/- 11.4 Mean ± SD	4436<br>9973	100%<br>100%	0.277	0.020
1<br>2	Male	2490<br>6501	56.1%<br>65.2%	<0.001	0.186
1<br>2	Female	1880<br>3363	42.4%<br>33.7%	<0.001	0.179
1<br>2	Unknown Gender	66<br>109	1.5%<br>1.1%	0.046	0.035
1<br>2	White	2892<br>6494	65.2%<br>65.1%	0.928	0.002
1<br>2	Black or African American ethnicity	708<br>1501	16.0%<br>15.1%	0.162	0.025
1<br>2	Unknown ethnicity	502<br>1241	11.3%<br>12.4%	0.055	0.035
1<br>2	Other ethnicity	207<br>447	4.7%<br>4.5%	0.624	0.009
1<br>2	Asian ethnicity	84<br>224	1.9%<br>2.2%	0.177	0.025
1<br>2	American Indian or Alaska Native ethnicity	34<br>44	0.8%<br>0.4%	0.014	0.042
1<br>2	Native Hawaiian or Other Pacific Islander	10<br>22	0.2%<br>0.2%	0.955	0.001
1<br>2	R56.9 Unspecified convulsions	1668<br>3633	37.6%<br>36.4%	0.024	0.024
1<br>2	G40.3 Generalized idiopathic epilepsy and epileptic syndromes	708<br>2114	16.0%<br>21.2%	0.135	0.135
1<br>2	G40.4 Other generalized epilepsy and epileptic syndromes	547<br>1341	12.3%<br>13.4%	0.033	0.033
1<br>2	G40.A Absence epileptic syndrome	122<br>362	2.8%<br>3.6%	0.050	0.050
1<br>2	G40.B Juvenile myoclonic epilepsy [impulsive petit mal]	106<br>261	2.4%<br>2.6%	0.015	0.015
1<br>2	G40.2 Localization-related (focal) (partial) symptomatic epilepsy and epileptic syndromes with complex partial seizures	756<br>1723	17.0%<br>17.3%	0.006	0.006
1<br>2	G40.1 Localization-related (focal) (partial) symptomatic epilepsy and epileptic syndromes with simple partial seizures	504<br>1054	11.4%<br>10.6%	0.025	0.025
1<br>2	G40.8 Other epilepsy and recurrent seizures	419<br>1086	9.4%<br>10.9%	0.048	0.048
1<br>2	G40.5 Epileptic seizures related to external causes	27<br>79	0.6%<br>0.8%	0.022	0.022
1<br>2	G40.0 Localization-related (focal) (partial) idiopathic epilepsy and epileptic syndromes with seizures of localized onset	250<br>526	5.6%<br>5.3%	0.016	0.016
1<br>2	F30-F39 Mood [affective] disorders	990<br>1623	22.3%<br>16.3%	0.154	0.154
1<br>2	F20-F29 Schizophrenia, schizotypal, delusional, and other non-mood psychotic disorders	255<br>451	5.7%<br>4.5%	0.056	0.056
1<br>2	F99-F99 Unspecified mental disorder (F99)	54<br>100	1.2%<br>1.0%	0.020	0.020
1<br>2	X71-X83 Intentional self-harm	25<br>43	0.6%<br>0.4%	0.019	0.019
1<br>2	W00-W19 Slipping, tripping, stumbling and falls	185<br>353	4.2%<br>3.5%	0.033	0.033
1<br>2	T14.91 Suicide attempt	26<br>31	0.6%<br>0.3%	0.041	0.041
1<br>2	1013711 Emergency Department Services	1167<br>2124	26.3%<br>21.3%	0.118	0.118
1<br>2	1013659 Hospital Inpatient and Observation Care Services	724<br>1126	16.3%<br>11.3%	0.146	0.146

Table S2: Co-prescribed antiseizure medications before and after propensity score matching – men and women combined

		**Characteristics before propensity score matching**				**Characteristics after propensity score matching**			
		Withdrawn from (n = 4,436) and remaining on (n = 9,973) valproate				Withdrawn from (n = 4,396) and remaining on (n = 4,396) valproate			
Cohort		Patients	% of Cohort	P-Value	Std diff.	Patients	% of Cohort	P-Value	Std diff.
1	lamotrigine	675	15.2%	0.002	0.056	662	15.1%	0.251	0.024
2		1,321	13.2%			624	14.2%		
1	topiramate	513	11.6%	<0.001	0.065	494	11.2%	0.148	0.031
2		954	9.6%			452	10.3%		
1	gabapentin	420	9.5%	<0.001	0.125	399	9.1%	0.291	0.023
2		611	6.1%			371	8.4%		
1	lacosamide	478	10.8%	<0.001	0.102	460	10.5%	0.215	0.026
2		779	7.8%			425	9.7%		
1	oxcarbazepine	310	7.0%	0.007	0.047	304	6.9%	0.284	0.023
2		581	5.8%			279	6.3%		
1	zonisamide	356	8.0%	0.102	0.029	349	7.9%	0.424	0.017
2		723	7.2%			329	7.5%		
1	phenytoin	168	3.8%	0.006	0.048	160	3.6%	0.730	0.007
2		291	2.9%			154	3.5%		
1	clobazam	401	9.0%	0.899	0.002	395	9.0%	0.306	0.022
2		895	9.0%			368	8.4%		
1	carbamazepine	159	3.6%	0.091	0.031	154	3.5%	0.516	0.014
2		417	4.2%			143	3.3%		
1	fosphenytoin	87	2.0%	<0.001	0.063	82	1.9%	0.811	0.005
2		118	1.2%			79	1.8%		
1	rufinamide	101	2.3%	0.015	0.045	101	2.3%	0.512	0.014
2		299	3.0%			92	2.1%		
1	ethosuximide	78	1.8%	0.172	0.024	78	1.8%	0.807	0.005
2		145	1.5%			75	1.7%		
1	felbamate	69	1.6%	0.348	0.017	67	1.5%	0.234	0.025
2		177	1.8%			54	1.2%		
1	perampanel	73	1.6%	0.267	0.020	71	1.6%	0.798	0.005
2		140	1.4%			68	1.5%		
1	primidone	17	0.4%	0.622	0.009	17	0.4%	0.177	0.029
2		33	0.3%			10	0.2%		
1	eslicarbazepine	39	0.9%	0.010	0.044	36	0.8%	0.810	0.005
2		51	0.5%			34	0.8%		
1	tiagabine	10	0.2%	0.094	0.028	10	0.2%	1	<0.001
2		11	0.1%			10	0.2%		
1	brivaracetam	64	1.4%	0.180	0.024	62	1.4%	0.657	0.009
2		117	1.2%			67	1.5%		
1	vigabatrin	10	0.2%	0.030	0.042	10	0.2%	1	<0.001
2		47	0.5%			10	0.2%		
1	methsuximide	10	0.2%	0.062	0.031	10	0.2%	1	<0.001
2		10	0.1%			10	0.2%		
1	cannabidiol	10	0.2%	0.062	0.031	10	0.2%	1	<0.001
2		10	0.1%			10	0.2%		
1	paramethadione	0	0%	--	--	0	0%	--	--
2		0	0%			0	0%		
1	lorazepam	1,061	23.9%	<0.001	0.121	1,032	23.5%	0.134	0.032
2		1,891	19.0%			973	22.1%		
1	diazepam	660	14.9%	0.191	0.024	651	14.8%	0.062	0.040
2		1,569	15.7%			590	13.4%		
1	midazolam	680	15.3%	<0.001	0.105	655	14.9%	0.202	0.027
2		1,171	11.7%			613	13.9%		
1	clonazepam	589	13.3%	0.210	0.023	581	13.2%	0.077	0.038
2		1,402	14.1%			526	12.0%		
1	levetiracetam	1,179	26.6%	<0.001	0.084	1,152	26.2%	0.272	0.023
2		2,289	23.0%			1,107	25.2%		
1	phenobarbital	120	2.7%	0.032	0.038	114	2.6%	0.790	0.006
2		212	2.1%			118	2.7%		

Table S3: Baseline characteristics before propensity score matching – men alone

	2490 withdrawn from valproate (cohort 1) and 6501 remaining on valproate (cohort 2)				
**Cohort**	**Characteristic**	**Patients**	**% of Cohort**	**P-Value**	**Std diff.**
1<br>2	Age at index 26.5 +/- 11.3 Mean ± SD<br>26.6 +/- 11.2 Mean ± SD	2490<br>6501	100%<br>100%	0.878	0.004
1<br>2	White	1647<br>4283	66.1%<br>65.9%	0.814	0.006
1<br>2	Black or African American ethnicity	408<br>999	16.4%<br>15.4%	0.234	0.028
1<br>2	Unknown ethnicity	246<br>712	9.9%<br>11.0%	0.140	0.035
1<br>2	Other ethnicity	123<br>318	4.9%<br>4.9%	0.925	0.002
1<br>2	Asian ethnicity	42<br>142	1.7%<br>2.2%	0.136	0.036
1<br>2	American Indian or Alaska Native ethnicity	17<br>31	0.7%<br>0.5%	0.231	0.027
1<br>2	Native Hawaiian or Other Pacific Islander	10<br>16	0.4%<br>0.2%	0.219	0.027
1<br>2	R56.9 Unspecified convulsions	921<br>2357	37.0%<br>36.3%	0.519	0.015
1<br>2	G40.3 Generalized idiopathic epilepsy and epileptic syndromes	398<br>1347	16.0%<br>20.7%	<0.001	0.123
1<br>2	G40.4 Other generalized epilepsy and epileptic syndromes	308<br>834	12.4%<br>12.8%	0.558	0.014
1<br>2	G40.A Absence epileptic syndrome	58<br>218	2.3%<br>3.4%	0.012	0.062
1<br>2	G40.B Juvenile myoclonic epilepsy [impulsive petit mal]	54<br>161	2.2%<br>2.5%	0.393	0.020
1<br>2	G40.2 Localization-related (focal) (partial) symptomatic epilepsy and epileptic syndromes with complex partial seizures	452<br>1154	18.2%<br>17.8%	0.656	0.010
1<br>2	G40.1 Localization-related (focal) (partial) symptomatic epilepsy and epileptic syndromes with simple partial seizures	306<br>736	12.3%<br>11.3%	0.200	0.030
1<br>2	G40.8 Other epilepsy and recurrent seizures	240<br>684	9.6%<br>10.5%	0.217	0.029
1<br>2	G40.5 Epileptic seizures related to external causes	10<br>41	0.4%<br>0.6%	0.196	0.032
1<br>2	G40.0 Localization-related (focal) (partial) idiopathic epilepsy and epileptic syndromes with seizures of localized onset	158<br>365	6.3%<br>5.6%	0.185	0.031
1<br>2	F30-F39 Mood [affective] disorders	463<br>945	18.6%<br>14.5%	<0.001	0.109
1<br>2	F20-F29 Schizophrenia, schizotypal, delusional, and other non-mood psychotic disorders	127<br>284	5.1%<br>4.4%	0.137	0.034
1<br>2	F99-F99 Unspecified mental disorder (F99)	28<br>69	1.1%<br>1.1%	0.795	0.006
1<br>2	X71-X83 Intentional self-harm	12<br>26	0.5%<br>0.4%	0.592	0.012
1<br>2	W00-W19 Slipping, tripping, stumbling and falls	98<br>208	3.9%<br>3.2%	0.085	0.040
1<br>2	T14.91 Suicide attempt	11<br>18	0.4%<br>0.3%	0.217	0.028
1<br>2	1013711 Emergency Department Services	622<br>1344	25.0%<br>20.7%	<0.001	0.103
1<br>2	1013659 Hospital Inpatient and Observation Care Services	395<br>710	15.9%<br>10.9%	<0.001	0.145

Table S4: Co-prescribed antiseizure medications before and after propensity score matching – men alone

**Characteristics before propensity score matching**						**Characteristics after propensity score matching**			
Men withdrawn from (n = 2,490) and remaining on valproate (n = 6,501)						Men withdrawn from (n = 2,482) and remaining on valproate (n = 2,482)			
Cohort		Patients	% of Cohort	P-Value	Std diff.	Patients	% of Cohort	P-Value	Std diff.
1<br>2	lamotrigine	342<br>823	13.7%<br>12.7%	0.174	0.032	338<br>318	13.6%<br>12.8%	0.402	0.024
1<br>2	topiramate	210<br>508	8.4%<br>7.8%	0.332	0.023	206<br>187	8.3%<br>7.5%	0.318	0.028
1<br>2	gabapentin	182<br>324	7.3%<br>5.0%	<0.001	0.097	180<br>173	7.3%<br>7.0%	0.699	0.011
1<br>2	lacosamide	287<br>523	11.5%<br>8.0%	<0.001	0.117	282<br>263	11.4%<br>10.6%	0.388	0.024
1<br>2	oxcarbazepine	179<br>387	7.2%<br>6.0%	0.031	0.050	177<br>174	7.1%<br>7.0%	0.868	0.005
1<br>2	zonisamide	184<br>435	7.4%<br>6.7%	0.242	0.027	181<br>176	7.3%<br>7.1%	0.784	0.008
1<br>2	phenytoin	103<br>192	4.1%<br>3.0%	0.005	0.064	99<br>83	4.0%<br>3.3%	0.227	0.034
1<br>2	clobazam	216<br>527	8.7%<br>8.1%	0.381	0.020	213<br>203	8.6%<br>8.2%	0.608	0.015
1<br>2	carbamazepine	81<br>273	3.3%<br>4.2%	0.039	0.050	81<br>76	3.3%<br>3.1%	0.685	0.012
1<br>2	fosphenytoin	50<br>77	2.0%<br>1.2%	0.003	0.066	47<br>41	1.9%<br>1.7%	0.519	0.018
1<br>2	rufinamide	60<br>189	2.4%<br>2.9%	0.198	0.031	60<br>51	2.4%<br>2.1%	0.388	0.025
1<br>2	ethosuximide	33<br>65	1.3%<br>1.0%	0.184	0.030	33<br>34	1.3%<br>1.4%	0.902	0.003
1<br>2	felbamate	39<br>111	1.6%<br>1.7%	0.640	0.011	39<br>34	1.6%<br>1.4%	0.555	0.017
1<br>2	perampanel	45<br>77	1.8%<br>1.2%	0.022	0.051	44<br>38	1.8%<br>1.5%	0.504	0.019
1<br>2	primidone	10<br>22	0.4%<br>0.3%	0.653	0.010	10<br>10	0.4%<br>0.4%	1	<0.001
1<br>2	eslicarbazepine	20<br>39	0.8%<br>0.6%	0.285	0.024	20<br>21	0.8%<br>0.8%	0.875	0.004
1<br>2	tiagabine	10<br>10	0.4%<br>0.2%	0.026	0.047	10<br>0	0.4%<br>0%	0.002	0.090
1<br>2	brivaracetam	36<br>72	1.4%<br>1.1%	0.188	0.030	36<br>32	1.5%<br>1.3%	0.625	0.014
1<br>2	vigabatrin	10<br>26	0.4%<br>0.4%	0.991	<0.001	10<br>10	0.4%<br>0.4%	1	<0.001
1<br>2	methsuximide	10<br>10	0.4%<br>0.2%	0.026	0.047	10<br>10	0.4%<br>0.4%	1	<0.001
1<br>2	cannabidiol	10<br>10	0.4%<br>0.2%	0.026	0.047	10<br>10	0.4%<br>0.4%	1	<0.001
1<br>2	paramethadione	0<br>0	0%<br>0%	--	--	0<br>0	0%<br>0%	--	--
1<br>2	lorazepam	595<br>1,182	23.9%<br>18.2%	<0.001	0.141	589<br>543	23.7%<br>21.9%	0.120	0.044
1<br>2	diazepam	359<br>1,007	14.4%<br>15.5%	0.205	0.030	357<br>333	14.4%<br>13.4%	0.325	0.028
1<br>2	midazolam	377<br>703	15.1%<br>10.8%	<0.001	0.129	371<br>334	14.9%<br>13.5%	0.132	0.043
1<br>2	clonazepam	297<br>881	11.9%<br>13.6%	0.041	0.049	295<br>267	11.9%<br>10.8%	0.210	0.036
1<br>2	levetiracetam	635<br>1,449	25.5%<br>22.3%	0.001	0.075	630<br>591	25.4%<br>23.8%	0.199	0.036
1<br>2	phenobarbital	75<br>134	3.0%<br>2.1%	0.007	0.060	73<br>66	2.9%<br>2.7%	0.547	0.017

Table S5: Baseline characteristics before propensity score matching – women alone

	1880 withdrawn from valproate (cohort 1) and 3363 remaining on valproate (cohort 2)				
**Cohort**	**Characteristic**	**Patients**	**% of Cohort**	**P-Value**	**Std diff.**
1<br>2	Age at index 28.4 +/- 11.8 Mean ± SD<br>28.3 +/- 11.9 Mean ± SD	1,880<br>3,363	100%<br>100%	0.711	0.011
1<br>2	White	1,207<br>1,206	66.6%<br>66.6%	0.972	0.001
1<br>2	Black or African American ethnicity	281<br>297	15.5%<br>16.4%	0.468	0.024
1<br>2	Unknown ethnicity	189<br>191	10.4%<br>10.5%	0.914	0.004
1<br>2	Other ethnicity	80<br>72	4.4%<br>4.0%	0.507	0.022
1<br>2	Asian ethnicity	41<br>36	2.3%<br>2.0%	0.565	0.019
1<br>2	American Indian or Alaska Native ethnicity	12<br>10	0.7%<br>0.6%	0.669	0.014
1<br>2	Native Hawaiian or Other Pacific Islander	10<br>0	0.6%<br>0%	0.002	0.105
1<br>2	R56.9 Unspecified convulsions	684<br>653	37.7%<br>36.0%	0.286	0.035
1<br>2	G40.3 Generalized idiopathic epilepsy and epileptic syndromes	285<br>269	15.7%<br>14.8%	0.460	0.025
1<br>2	G40.4 Other generalized epilepsy and epileptic syndromes	221<br>212	12.2%<br>11.7%	0.645	0.015
1<br>2	G40.A Absence epileptic syndrome	61<br>52	3.4%<br>2.9%	0.390	0.029
1<br>2	G40.B Juvenile myoclonic epilepsy [impulsive petit mal]	47<br>37	2.6%<br>2.0%	0.270	0.037
1<br>2	G40.2 Localization-related (focal) (partial) symptomatic epilepsy and epileptic syndromes with complex partial seizures	273<br>248	15.1%<br>13.7%	0.237	0.039
1<br>2	G40.1 Localization-related (focal) (partial) symptomatic epilepsy and epileptic syndromes with simple partial seizures	183<br>168	10.1%<br>9.3%	0.400	0.028
1<br>2	G40.8 Other epilepsy and recurrent seizures	169<br>156	9.3%<br>8.6%	0.450	0.025
1<br>2	G40.5 Epileptic seizures related to external causes	15<br>16	0.8%<br>0.9%	0.857	0.006
1<br>2	G40.0 Localization-related (focal) (partial) idiopathic epilepsy and epileptic syndromes with seizures of localized onset	85<br>77	4.7%<br>4.2%	0.520	0.021
1<br>2	F30-F39 Mood [affective] disorders	462<br>463	25.5%<br>25.6%	0.970	0.001
1<br>2	F20-F29 Schizophrenia, schizotypal, delusional, and other non-mood psychotic disorders	112<br>114	6.2%<br>6.3%	0.891	0.005
1<br>2	F99-F99 Unspecified mental disorder (F99)	23<br>24	1.3%<br>1.3%	0.883	0.005
1<br>2	X71-X83 Intentional self-harm	12<br>10	0.7%<br>0.6%	0.669	0.014
1<br>2	W00-W19 Slipping, tripping, stumbling and falls	75<br>76	4.1%<br>4.2%	0.934	0.003
1<br>2	T14.91 Suicide attempt	11<br>12	0.6%<br>0.7%	0.834	0.007
1<br>2	1013711 Emergency Department Services	489<br>480	27.0%<br>26.5%	0.736	0.011
1<br>2	1013659 Hospital Inpatient and Observation Care Services	277<br>264	15.3%<br>14.6%	0.545	0.020

Table S6: Co-prescribed antiseizure medications before and after propensity score matching – women alone

Characteristics before propensity score matching						Characteristics after propensity score matching			
Women withdrawn from (n = 1,880) and remaining on valproate (n = 3,363)						Women withdrawn from (n = 1,812) and remaining on valproate (n = 1,812)			
Cohort		Patients	% of Cohort	P-Value	Std diff	Patients	% of Cohort	P-Value	Std diff
1<br>2	lamotrigine	326<br>481	17.3%<br>14.3%	0.003	0.083	308<br>297	17.0%<br>16.4%	0.624	0.016
1<br>2	topiramate	290<br>427	15.4%<br>12.7%	0.006	0.079	265<br>252	14.6%<br>13.9%	0.537	0.021
1<br>2	gabapentin	234<br>277	12.4%<br>8.2%	<0.001	0.139	202<br>207	11.1%<br>11.4%	0.793	0.009
1<br>2	lacosamide	180<br>248	9.6%<br>7.4%	0.005	0.079	161<br>149	8.9%<br>8.2%	0.476	0.024
1<br>2	oxcarbazepine	127<br>189	6.8%<br>5.6%	0.098	0.047	116<br>96	6.4%<br>5.3%	0.157	0.047
1<br>2	zonisamide	170<br>280	9.0%<br>8.3%	0.374	0.025	159<br>140	8.8%<br>7.7%	0.251	0.038
1<br>2	phenytoin	61<br>96	3.2%<br>2.9%	0.427	0.023	56<br>59	3.1%<br>3.3%	0.776	0.009
1<br>2	clobazam	183<br>359	9.7%<br>10.7%	0.283	0.031	176<br>158	9.7%<br>8.7%	0.301	0.034
1<br>2	carbamazepine	76<br>139	4.0%<br>4.1%	0.874	0.005	71<br>65	3.9%<br>3.6%	0.600	0.017
1<br>2	fosphenytoin	37<br>41	2.0%<br>1.2%	0.032	0.060	30<br>28	1.7%<br>1.5%	0.791	0.009
1<br>2	rufinamide	41<br>105	2.2%<br>3.1%	0.047	0.059	40<br>42	2.2%<br>2.3%	0.823	0.007
1<br>2	ethosuximide	43<br>78	2.3%<br>2.3%	0.941	0.002	41<br>43	2.3%<br>2.4%	0.825	0.007
1<br>2	felbamate	28<br>66	1.5%<br>2.0%	0.216	0.036	25<br>26	1.4%<br>1.4%	0.888	0.005
1<br>2	perampanel	28<br>59	1.5%<br>1.8%	0.471	0.021	26<br>21	1.4%<br>1.2%	0.463	0.024
1<br>2	primidone	10<br>11	0.5%<br>0.3%	0.260	0.031	10<br>10	0.6%<br>0.6%	1	<0.001
1<br>2	eslicarbazepine	18<br>12	1.0%<br>0.4%	0.006	0.074	10<br>11	0.6%<br>0.6%	0.827	0.007
1<br>2	tiagabine	0<br>10	0%<br>0.3%	0.018	0.077	0<br>0	0%<br>0%	--	--
1<br>2	brivaracetam	27<br>44	1.4%<br>1.3%	0.701	0.011	25<br>22	1.4%<br>1.2%	0.660	0.015
1<br>2	vigabatrin	0<br>21	0%<br>0.6%	0.001	0.112	0<br>0	0%<br>0%	--	--
1<br>2	methsuximide	0<br>10	0%<br>0.3%	0.018	0.077	0<br>0	0%<br>0%	--	--
1<br>2	cannabidiol	10<br>0	0.5%<br>0%	<0.001	0.103	0<br>0	0%<br>0%	--	--
1<br>2	paramethadione	0<br>0	0%<br>0%	--	--	0<br>0	0%<br>0%	--	--
1<br>2	lorazepam	457<br>692	24.3%<br>20.6%	0.002	0.090	415<br>414	22.9%<br>22.8%	0.968	0.001
1<br>2	diazepam	289<br>545	15.4%<br>16.2%	0.429	0.023	278<br>269	15.3%<br>14.8%	0.676	0.014
1<br>2	midazolam	292<br>451	15.5%<br>13.4%	0.035	0.060	266<br>254	14.7%<br>14.0%	0.570	0.019
1<br>2	clonazepam	284<br>507	15.1%<br>15.1%	0.976	0.001	271<br>265	15.0%<br>14.6%	0.779	0.009
1<br>2	levetiracetam	524<br>811	27.9%<br>24.1%	0.003	0.086	494<br>483	27.3%<br>26.7%	0.681	0.014
1<br>2	phenobarbital	43<br>78	2.3%<br>2.3%	0.941	0.002	39<br>39	2.2%<br>2.2%	1	<0.001

Table S7: Competing Risks Results Table – men and women combined

Outcome Name	Cohort Statistics		
	Patient Count	Percentage of Cohort	Cumulative Incidence at End of Time Window
**Withdrawn from valproate**			
≥1 seizure-coded healthcare consultations	1618	36.5%	0.3995
≥1 emergency department attendances	584	13.2%	0.1449
≥1 injuries	453	10.2%	0.1119
≥1 hospital admissions	406	9.2%	0.1017
New-onset depression	315	7.1%	0.0781
≥1 falls	70	1.6%	0.0174
All-cause death	43	1.0%	0.0109
≥1 aspiration pneumonia infections	31	0.7%	0.0077
≥1 Self-harm or suicide attempts	10	0.2%	0.0002
**Remaining on valproate**			
≥1 seizure-coded healthcare consultations	3935	39.5%	0.4248
≥1 emergency department attendances	1043	10.5%	0.1130
≥1 injuries	982	9.8%	0.1074
≥1 hospital admissions	817	8.2%	0.0876
New-onset depression	543	5.4%	0.0581
≥1 falls	143	1.4%	0.0155
All-cause death	90	0.9%	0.0104
≥1 aspiration pneumonia infections	95	1.0%	0.0102
≥1 Self-harm or suicide attempts	10	0.1%	0.0002
